# Sex Differences in Resting Metabolic Rate among Athletes and Association with Body Composition Parameters: A Follow-Up Investigation

**DOI:** 10.3390/jfmk8030109

**Published:** 2023-08-03

**Authors:** Andrew R. Jagim, Margaret T. Jones, Andrew T. Askow, Joel Luedke, Jacob L. Erickson, Jennifer B. Fields, Chad M. Kerksick

**Affiliations:** 1 Sports Medicine, Mayo Clinic Health System, Onalaska, WI 54650, USA; jagim.andrew@mayo.edu (A.R.J.); luedke.joel@mayo.edu (J.L.); erickson.jacob@mayo.edu (J.L.E.); 2 Patriot Performance Laboratory, Frank Pettrone Center for Sports Performance, George Mason University, Fairfax, VA 22030, USA; jennifer.fields@uconn.edu; 3 Department of Exercise and Sport Science, University of Wisconsin-La Crosse, La Crosse, WI 54601, USA; 4 Sport, Recreation, and Tourism Management, George Mason University, Fairfax, VA 22030, USA; 5 Department of Kinesiology and Community Health, University of Illinois at Urbana-Champaign, Urbana, IL 61820, USA; askow2@illinois.edu; 6 Department of Nutritional Sciences, University of Connecticut, Storrs, CT 06269, USA; 7 Exercise and Performance Nutrition Laboratory, Department of Kinesiology, Lindenwood University, St. Charles, MO 63301, USA; ckerksick@lindenwood.edu

**Keywords:** resting metabolic rate, sex differences, metabolism, body composition, athletes

## Abstract

The purpose of this study was to examine sex differences in resting metabolic rate (RMR) and associations between measured RMR and body composition parameters in athletes. One-hundred and ninety collegiate men (n = 98; age: 20.1 ± 1.6 yr.; body mass: 92.7 ± 17.5 kg; height: 181.6 ± 6.2 cm, body mass index: 28.0 ± 4.7 kg/m^2^) and women (n = 92; age: 19.4 ± 1.1 yr.; body mass: 65.2 ± 11.0 kg; height: 168.0 ± 6.6 cm, body mass index: 23.0 ± 3.6 kg/m^2^) athletes volunteered to participate in this study. Athletes completed a body composition assessment using air displacement plethysmography and RMR using indirect calorimetry. Assessments were completed in a fasted state and after refraining from intense physical activity > 24 h prior to testing. Data were collected during the 2016–2019 seasons. Men had a higher RMR compared to women (2595 ± 433 vs. 1709 ± 308 kcals; *p* < 0.001); however, when adjusted for body mass (*p* = 0.064) and fat-free mass (*p* = 0.084), the observed differences were not significant. Height, body mass, body mass index, fat-free mass, and fat mass were positively associated with RMR in both men and women athletes (r = 0.4–0.8; *p* < 0.001). Body mass (men: β = 0.784; women: β = 0.832)) was the strongest predictor of RMR. Men athletes have a higher absolute RMR compared to their women counterparts, which is influenced by greater body mass and fat-free mass. Body mass is the strongest predictor of RMR in both men and women athletes.

## 1. Introduction

For athletes to ensure adequate energy intake for optimal performance, recovery, and health, maintaining energy balance is essential. In order to establish individualized energy recommendations for athletes, the total daily energy expenditure must be determined. Resting metabolic rate (RMR) accounts for nearly 60–70% of total daily energy expenditure; thus, determining RMR serves as an essential part of identifying an athlete’s energy requirement. 

RMR is largely influenced by body size and body composition as metabolic activity may vary across different tissue compartments, thereby influencing energy expenditure [1,2,3]. Further, previous research has proposed sex as a contributing factor of RMR, regardless of age [4,5,6,7], with men displaying ~23% higher RMR values compared to women, even after controlling for fat-free mass [7]. Less is known regarding potential sex differences in RMR among athletic populations. Previously [5], our group reported notable differences in RMR between men and women athletes among a mixed cohort of collegiate athletes. However, when normalized to body mass and fat-free mass, there were no longer differences observed for RMR, indicating that body size differences accounted for variations in absolute measures of RMR. However, more work is needed with a larger sample size to confirm these preliminary findings. Upon further analysis, it was found that body mass was the biggest predictor of RMR in both the men and women athlete sub-groups, accounting for 53 and 77% of the variation in measured RMR, respectively [5]. Conversely, previous work has proposed that fat-free mass is the largest contributor to RMR [8,9]. The varying degrees of influence on RMR from body composition parameters may have important implications for weight loss interventions [10], body composition goals [11], and the selection of appropriate RMR-prediction equations [1].

Further evaluation of sex differences in RMR among athlete populations is necessary to enable individualized energy prescription regarding nutritional recommendations for sports performance and overall health. This would allow for the development of sex-specific nutritional recommendations and provide additional insight as to how body size and composition may influence metabolic activity and the resulting energy requirement of athletes. This may help to identify appropriate RMR prediction equations when the option of directly measuring RMR is not readily available. Therefore, the primary aim of the current study was to examine sex differences in RMR among a large mixed cohort of athletes as a follow-up to our original study. A secondary aim was to examine predictors of RMR.

## 2. Methods

### 2.1. Subjects

One-hundred and ninety collegiate men (n = 98; age: 20.1 ± 1.6 yr.; body mass: 92.7 ± 17.5 kg; height: 181.6 ± 6.2 cm, body mass index: 28.0 ± 4.7 kg/m^2^) and women (n = 92; age: 19.4 ± 1.1 y.; body mass: 65.2 ± 11.0 kg; height: 168.0 ± 6.6 cm, body mass index: 23.0 ± 3.6 kg/m^2^) volunteered to participate in this study. All athletes were screened for health contraindications by the sports medicine staff as part of the team’s normal standard of care. Inclusion criteria included being medically cleared to participate on the team. Exclusion criteria included currently being treated for or diagnosed with a cardiac, respiratory, circulatory, autoimmune, musculoskeletal, metabolic, hematological, neurological, or endocrine disorder or disease. Male athletes competing in baseball: n = 8; cross country: n = 5; football: n = 62; track and field: n = 6; and wrestling: n = 17, with an average weekly training volume of 7.7 ± 4.7 h per week, and female athletes competing in cross country: n = 9; diving: n = 2; soccer: n = 43; swimming: n = 4; track: n = 23; volleyball: n = 9; and tennis: n = 2, with an average weekly training volume of 9.4 ± 3.1 h per week, participated in the current study. Interested athletes were informed of the risks associated with the study, and they provided their written informed consent prior to participation. All procedures involving human subjects were conducted in accordance with the requirements of the Declaration of Helsinki and approved by an Institutional Review Board. Trained research personnel conducted all study data collection according to standard laboratory practices.

### 2.2. Study Design

Data were collected during the 2016–2019 competitive sport seasons. Athletes completed testing during the pre-season period (within 6 weeks of the competitive season). During a single morning of testing between the hours of 0600 and 1000 with each testing session lasting approximately 1 h, the athletes completed a body composition assessment and RMR test in a climate-controlled laboratory setting (temperature range: 22–24 °C and relative humidity range: 37–44%). Participants reported to the laboratory in a fasted (>12 h) state and were advised to consume 0.5 L of water upon waking, but no later than within 1 h. of testing. Participants were also asked to refrain from strenuous activity <24 h prior to testing. This included strength training activities, conditioning, and sport-specific practice sessions greater than 50% effort.

### 2.3. Data Collection Procedures

#### 2.3.1. Body Composition

Height and body mass were assessed with a Seca^®^ physicians scale and stadiometer. Participants then completed a body composition assessment via air displacement plethysmography (BODPOD, Cosmed, Chicago, IL, USA). Calibration procedures were completed in accordance with the manufacturer guidelines using a calibration cylinder of a standard volume (49.55 L) and an empty chamber prior to testing for the day. Participants wore tight-fitting clothing, removed all jewelry, and wore a swim cap prior to entering the testing chamber. Thoracic gas volumes were predicted using manufacturer default settings. Fat mass and fat-free mass (FFM) were determined based on the participant’s body mass and measured body volume using the Brozek equation [12]. Test to test reliability of performing this body composition assessment in our lab with athletic populations has yielded high reliability for BM (r = 0.999), body fat percent (0.994), and FFM (0.998).

#### 2.3.2. Resting Metabolic Rate

Indirect calorimetry (ParvoMedics True One Metabolic System, Salt Lake City, UT, USA) was used for RMR measurement. Participants were instructed to remain motionless in a supine position on an examination table. A clear, hard plastic hood and soft, clear plastic drape was then placed over the participant’s neck, head, and shoulders to determine resting oxygen uptake and energy expenditure. Participants were instructed to remain awake throughout the duration of testing. Data were recorded after the first ten minutes of testing during a five-minute period in which criterion variables (e.g., VO_2_ L/min) did not vary by more than 5%.

### 2.4. Statistical Analyses

One-way analysis of variance was used to examine sex differences in RMR. Absolute measures of RMR were then normalized to body mass and fat-free mass. The relationship between RMR and body composition parameters was assessed using linear regression. Stepwise multiple linear regression analyses were used to determine which predictor variables (body mass, body mass index, fat-free mass, body fat %, fat mass) best predicted RMR in men and women. Slope was calculated to determine the observed changes in RMR per 1 unit increase in body mass and fat-free mass. The standard error of estimate (SEE) was determined to help evaluate the fit of the regression model for the measured RMR values. Normality was assessed via a visual inspection of normal Q-Q plots and skewness/kurtosis values. Homoscedasticity was assessed with Levene’s Test of Equality of Error Variances. Statistical analyses were conducted using the IBM SPSS Statistics for Windows (version 25.0; IBM Corp., Armonk, NY, USA). Strength of correlation coefficients was classified as trivial (|r| < 0.10), weak (0.10 ≤ |r| < 0.30), moderate (0.30 ≤ |r| < 0.50), strong (0.50 ≤ |r| < 0.70), very strong (0.70 ≤ |r| < 0.90), and nearly perfect (r ≥ 0.90) [13].

## 3. Results

Men were taller and had a greater body mass, body mass index, fat-free mass, and a lower body fat percentage (*p* < 0.001) (Table 1).

**Figure 1:**
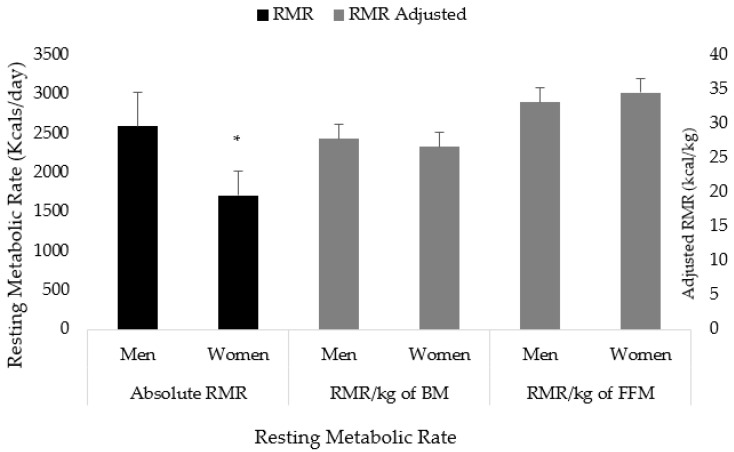
Sex differences in resting metabolic rate. RMR = resting metabolic rate; BM = body mass; FFM = fat-free mass; kg = kilograms. * Denotes *p* < 0.05.

Men had a higher RMR compared to women (2595 ± 433 vs. 1709 ± 308 kcals; *p* < 0.001). When adjusted for body mass (*p* = 0.064) and fat-free mass (*p* = 0.084), the observed differences were no longer significant (Figure 1).

**Figure 2:**
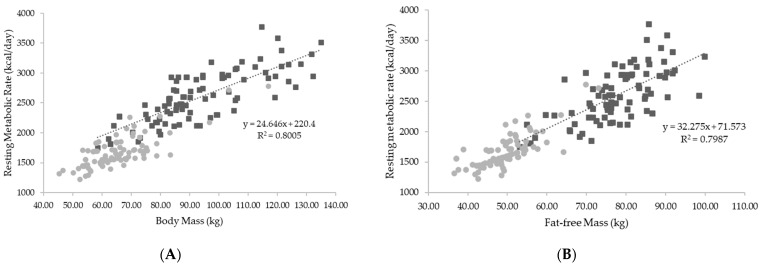
(**A**) Relationships between body mass and resting metabolic rate; (**B**) relationship between fat-free mass and resting metabolic rate. Kcal = kilocalories; kg = kilograms. Gray circles represent female athletes; black squares represent male athletes.

Body mass and fat-free mass were the strongest predictors of RMR. Figure 2 represents the relationship between body mass, fat-free mass, and RMR for all athletes combined.

In men, there was a very strong relationship between body mass (r = 0.78), body mass index (r = 0.72), and RMR. Fat-free mass, body fat percentage, and fat mass were strongly associated with RMR in men (r = 0.53–0.67; *p* < 0.001), and there was a moderate relationship between height and RMR (r = 0.40). In men, body mass (β = 0.784) was the strongest predictor of RMR (Table 2).

In women, there was a very strong relationship between body mass (r = 0.83), fat-free mass (r = 0.77), and RMR. Body mass index and fat mass were strongly associated with RMR in women (r = 0.69), and there was a moderate relationship between height, body fat percentage, and RMR (r = 0.48–0.49). In women, body mass (β = 0.832) was the strongest predictor of RMR (Table 3).

## 4. Discussion

The primary aim of the current study was to examine sex differences in RMR among a large mixed cohort of men and women collegiate athletes. To the best of our knowledge, this is the largest study to date evaluating sex-specific differences in RMR among collegiate athletes. The main findings from the current study indicate that men athletes have a higher RMR, which is largely influenced by their larger body stature, specifically greater body mass when compared to their female counterparts. On average, males had a 50% higher RMR compared to females. However, when RMR was normalized to body mass and fat-free mass, the differences in RMR were no longer significant (Figure 1). This is in alignment with our previous findings in NCAA Division III collegiate athletes [5], and previous research in endurance [14] and adolescent athletes [2], yet contradictory to research in older adult (non-athlete) populations [4,6,7]. For example, we previously reported significantly greater RMR values in male athletes compared to females; similarly, when normalized to body mass and fat-free mass, sex differences were no longer observed [5]. Thompson and Manore [14] also found male athletes to have higher RMR values than female athletes (i.e., males: 1868 ± 239 vs. females: 1486 ± 103 kcal/day) among a cohort of highly trained endurance athletes. The observed similarities in the study by Manore and Thompson are noteworthy as the RMR values of the endurance athletes were notably lower than those from the current study, likely a result of the lower body mass and FFM values of the endurance athletes compared to the mixed cohort of athletes in the current study. Despite the differences in body size and RMR, the resulting RMR values after normalizing to FFM were similar in both studies, suggesting that sex differences in body mass and FFM and the subsequent difference in RMR may be similar across different types of athletes. This relationship also appears to be evident among younger athletes as similar findings were reported by Reale et al. [2] among a diverse sample of adolescent athletes where males exhibited higher absolute measures of RMR; however, when normalized to body mass and fat-free mass, RMR values were equivocal, albeit significance testing was not performed.

A secondary aim of the current study was to identify predictors of RMR. The results demonstrated that body mass was the strongest predictor of RMR for both male and female athletes, which is in agreement with our initial findings [5] and those previously reported in elite-level national athletes [15], yet contradictory to previous reports [1,16,17,18,19]. In our initial study, it was reported that body mass accounted for 53% and 77% of the variation in measured RMR for men and women, respectively. In comparison, results from the current study found that body mass accounted for 78% and 83% of the variation in RMR for male and female athletes, respectively, which is an even stronger association than initially observed. In contrast, Watson et al. [3] found fat-free mass to be the strongest predictor of RMR among a sample of Division II female athletes. Similarly, among a sample of adolescent athletes, Reale et al. [2] also found fat-free mass to be the strongest predictor of RMR, accounting for 77% of the variation in RMR. However, body mass was a close second, accounting for 71% of RMR. The authors noted the value of body mass being able to predict RMR as body-mass-based RMR prediction equations would not require a body composition measurement to be completed when estimating an athlete’s RMR. In the current study, every 1 kg increase in body mass was associated with a 19 kcal and 20 kcal increase in RMR for male and female athletes, respectively. It is worth noting that this is not indicative of a direct increase in metabolic activity following increases in body mass at an individual level, which warrants further investigation to examine the specific increase in energy expenditure that may ensue. Alternatively, fat-fee mass-based equations have been found to best predict RMR among some athlete populations [1,3]. In the current study, fat-free mass was found to be the second-best predictor of RMR in male and female athletes, accounting for 67% and 77% of the variance in RMR, respectively. There is evidence for an equal ability of both fat-free mass and body-mass-based equations to predict RMR [20,21] among the same cohort of athletes. In the current study, every 1 kg increase in fat-free mass was associated with a 31 kcal and 34 kcal increase in RMR for male and female athletes, respectively. Although fat-free mass commonly serves as a significant predictor of acute measures of RMR in athletes, increases in fat-free mass do not always correspond to subsequent increases in RMR [22], indicating that meaningful increases in fat-free mass and overall body mass may be required to elicit practically meaningful increases in RMR over time. However, more work is warranted to elucidate how body mass and compositional changes over time may subsequently influence changes in RMR.

The mean RMR values of the male athletes included in the current study (2595 ± 433 kcals) are higher than those previously reported in male endurance (1868 ± 239 kcals) [14], highly trained (1858 kcals) [23], endurance trained (1808 kcals) [24], elite mixed sport (2099 ± 400 kcals) [15], and bodybuilding (2015 ± 457 kcals) athletes [20]. Similarly, the mean RMR values of the female athletes included in the current study were higher (1709 ± 308 kcals) than those previously reported in female NCAA Division II (1466 ± 150 kcals) [3], endurance (1486 ± 103 kcals) [14], elite mixed sport (1577 ± 170 kcals) [15], and professional dance (1215 ± 106 kcals) [25] athletes. Considering the activity levels of the athletes in the current study are likely comparable to those of athletes evaluated in previous studies, it is likely the higher body mass and FFM values may explain the higher RMR values of the athletes evaluated in the current study. Additionally, as both body mass and FFM were found to be significant predictors of RMR, higher values would correspond to higher measured RMR values. In support, Koshimizu et al. 2012 [26] observed higher RMR values in larger athletes competing in strength and power sports compared to smaller athletes competing in endurance sports.

This study is not without limitations. The current study was conducted in collegiate athletes; therefore, it is unknown if sex differences in RMR would exist, along with similar relationships between body composition and RMR, in untrained or overweight populations. Menstrual status was not standardized across female athletes during the data collection process. A detailed training load was not collected prior to testing. This information could have help evaluate relationships between acute and chronic workloads and how prior activity may influence RMR. Additionally, energy availability and dietary intake were not accounted for in the days and weeks prior to testing. Lastly, hormone levels were not evaluated, specifically catecholamine or sex hormone concentrations, which have been shown to influence RMR [4].

## 5. Conclusions

In NCAA Division III athletes, men have a higher absolute RMR than women. However, when normalized to body mass and fat-free mass, there are no differences in RMR between men and women, suggesting that male athletes tend to have a higher RMR because of their greater body mass. Additionally, body mass was the strongest predictor of RMR in both men and women athletes. Therefore, larger athletes have higher metabolic requirements than their smaller counterparts. These findings may help direct nutritional recommendations for athletes to help them meet their energy requirements. An emphasis should be place on a higher energy intake for larger athletes to ensure adequate energy availability to support their physiological requirements and demands of their sport activity.

## Figures and Tables

**Table 1:** Summary of subject characteristics.

Variable	Men	Women	*p* Value
Age (y.)	20.1 ± 1.6	19.5 ± 1.1	<0.001
Height (cm)	181.6 ± 6.2	168.0 ± 6.6	<0.001
Body mass (kg)	92.7 ± 17.5	65.2 ± 11.0	<0.001
Body mass index (kg/m^2^)	28.0 ± 4.7	23.0 ± 3.6	<0.001
Body fat (%)	15.6 ± 8.8	22.7 ± 6.0	<0.001
Fat-free mass (kg)	77.1 ± 9.4	49.6 ± 6.4	<0.001
Fat mass (kg)	15.3 ± 11.3	15.1 ± 9.2	0.884

Data presented as Mean ± SD.

**Table 2:** Regression analysis in men athletes.

Variable	R^2^	β	Slope	SEE	*p* Value
Height	0.163	0.404	27.34	6.53	<0.001
Body mass	0.615	0.784	19.20	1.60	<0.001
Body mass index	0.521	0.722	66.36	6.71	<0.001
Body Fat %	0.279	0.529	25.33	4.29	<0.001
Fat-free mass	0.455	0.674	31.11	3.59	<0.001
Fat mass	0.404	0.636	23.83	3.05	<0.001

**Table 3:** Regression analysis in women athletes.

Variable	R^2^	β	Slope	SEE	*p* Value
Height	0.244	0.494	21.02	4.66	<0.001
Body mass	0.692	0.832	20.96	1.76	<0.001
Body mass index	0.478	0.692	55.40	7.29	<0.001
Body fat %	0.229	0.479	24.95	5.76	<0.001
Fat-free mass	0.593	0.770	33.50	3.50	<0.001
Fat mass	0.489	0.699	31.54	4.06	<0.001

## Data Availability

Data are available upon request.
